# Real-Time Monitoring of Chemical Composition in Nickel-Based Laser Cladding Layer by Emission Spectroscopy Analysis

**DOI:** 10.3390/ma12162637

**Published:** 2019-08-19

**Authors:** Siyu Wang, Changsheng Liu

**Affiliations:** School of Material Science and Engineering, Northeastern University, Shenyang 110819, China

**Keywords:** real-time monitoring, emission spectroscopy, chemical composition, laser cladding, Nickel-based alloy

## Abstract

The composition distribution can influence the performances of laser cladding layers. Hence, the technology of rthe eal-time monitoring of chemical composition is required to apply on laser cladding process. In this experiment, four kinds of Ni-based alloy powders were used to prepare laser cladding layers on AISI (American Iron and Steel Institute) 4140 steel. At the same time, emission spectra were collected during real-time laser cladding process. The intensity of spectral lines were revised with a corrected number deduced with evaporation rate of elements. By correlating the weight ratios of elements with the intensity ratios of spectral lines, four calibration curves were established to monitor composition distribution. The main results are shown as following: Weight ratios among elements in the laser cladding layers changed versus input energy density due to different saturated vapor pressures among elements; the dilution amount of substrate showed weak relations under the different manufacturing parameters, and the main reason for this can be attributed to the change of thermo–physical properties among different Ni-based alloy powders; the predicted results showed that when the composition concentration was higher than 3 wt.%, the relative error was lower than 8%, compared with EDS (Energy-dispersive X-ray spectroscopy) testing data.

## 1. Introduction

The technology of laser cladding is capable of fabricating a wide range of surface alloys and composites of required properties. In addition, the alloy coating producing by laser cladding is also cost-effective for enabling the salvage of expensive parts, and the point light source with a high energy makes it possible to process the intractable alloy [1]. According to the characteristics of laser cladding, Nickel-based superalloys—with their many excellent properties [2], specific machinability and the expensive cost [3]—are widely applied on the fabrication of laser cladding layers to enhance the performances of the original surface of substrates [4,5,6,7,8]. However, according to the characteristics of laser cladding, the whole cladding layer is prepared line by line overlappingly, and the parts of substrate can be melted and diluted into the cladding layer during the process [1]. Thus, the composition in the cladding layer is different from the raw alloy powder, and the properties of the cladding layer can be directly influenced by the composition. To test the real composition in the cladding layer, the sample usually needs to be taken out from the completed layer in a destructive way. Thus, the technologies for both nondestructive testing and real-time monitoring are required to apply to laser cladding process.

Laser induced breakdown spectroscopy (LIBS) is a kind of emission spectroscopy applied to chemical analysis [9]. It can measure composition during interactions between a laser pulse and a material surface. Therefore, it has the potential to be a nondestructive method to monitor the composition of a laser cladding layer. There are many analysis methods used in the measurement of LIBS. By establishing calibration curves constructed by correlating the composition to intensity ratios among spectral lines, Aragon et al. [10] got a 1%–13% relative standard deviation of the concentration ratio versus 1.1%–2.9% relative standard deviation of the intensity ratio for several different elements with a content higher than 0.1%. Ciucci et al. [11] used a calibration-free method to complete the quantitative elemental analysis with the data of LIBS—by this method, the substrate effects were overcome, and the relative composition for high concentration elements was lower than 0.3%; however, for low concentration elements, the maximal relative composition variation was 38%. Death et al. [12] used LIBS in combination with principal components regression (PCR) to determine the elemental composition of iron ore samples—the average error for the calibration model and prediction results were both lower than 14%. Yaroshchyk et al. [13] created a theoretical emission spectrum based on a database of atomic emission lines by comparing observed and predicted spectra, and the concentration error of the majority of elements was lower than 25%. Moreover, for the application of emission spectroscopy in real-time laser cladding, Liu et al. [14] calculated the electron temperature in the plasma plume, and they found that the electron temperature can be an indicator of the instability of the process and clad quality. Song and Mazumder [15] used emission spectra to calculated element concentration in an Fe-Cr cladding layer—the accuracy of the measured Cr concentration was within 2.78 at.%, and the average resolution was around 5.21 at.%. Moreover, they found the phase transition in the cladding layer could make odd variations in the spectra [16]. Additionally, to improve the accuracy of the monitoring system used in real time laser processing, Song et al. also completed lots of work regarding the calculation method of calibration curves [17].

It should be pointed out that the laser energy density used for LIBS is much higher than that used for real-time laser manufacturing. Thus, the mechanism of phase transition is different from LIBS to real-time laser cladding. In traditional LIBS, the solid phase is directly ablated into the gas phase, and the composition is same in both the solid and gas phases. However, in the process of laser cladding, the solid phase is melted into the liquid phase, and then the liquid phase is evaporated into the gas phase. Due to the influence of saturated vapor pressure, the composition is different among these three states. Hence, the error of composition between the liquid phase and the gas phase must be revised during real time laser cladding. According to related reports, the analysis method used for real time composition monitoring was always cited by LIBS, but there is little research about the error revision of composition between the liqiud state and the gas state. Therefore, in this study, four kinds of Ni-based alloy powders were used to prepared the laser cladding layers. At the same time, the emission spectra were collected during the laser cladding process, and the spectral lines emitted by the atoms of substrate and alloy elements were selected in spectra. For compensating the composition deviation affected by partial pressure of the metal gas, a corrected parameter—which was deduced by real elements distribution in cladding layers—was used to resive the intensity of spectral lines. Moreover, the calibration curves were established by corelating the composition concentration with the intensity ratio of spectral lines. In the end, the prediction results analyzed by the monitoring system were compared with that measured by energy dispersive spectrometry (EDS).

## 2. Experiment and Method

### 2.1. Sample Preparation

**Figure 1:**
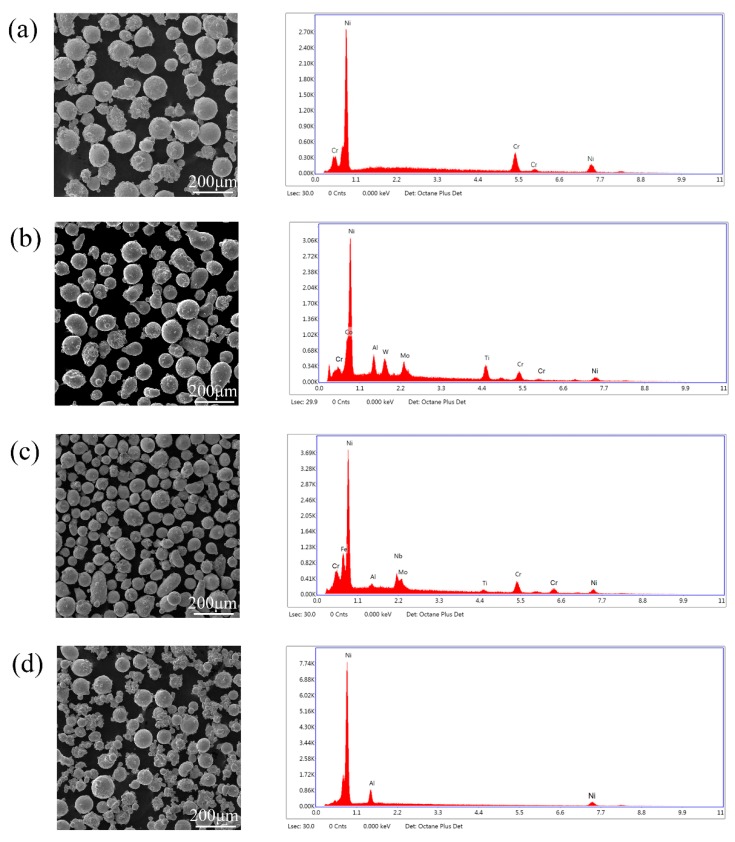
Morphology and energy dispersive spectrometry (EDS) results of Ni-based alloy powders. (**a**) Ni 107; (**b**) Ni 183; (**c**) Ni 202; (**d**) Ni 357.

In this experiment, 4 kinds of Ni-based alloy powders (Praxair Surface Technologies, Indianapolis, IN, USA) were used to fabricated cladding layers on AISI 4140 steel. The morphology and composition of Ni-based alloy powders were tested by scanning electron microscopy (SEM) and EDS (Tescan MIRA3 FEG SEM, Warrendale, PA, USA), and the tested results were shown in Figure 1 and Table 1. The substrate composition was shown in Table 2.

By comparing morphologies among Ni-based alloy powders, it could be observed that for all kinds of Ni-based alloy powders, the shape was irregular and the size was not uniform. Hence, the method of pre-placed powder layer was used in the experiment to avoid the bad influence from the defects of the morphology of alloy powder. The substrates were annealed before the experiment, and then they were cut into small size as 1 inch × 2 inch × 0.25 inch (5.08 cm × 2.54 cm × 0.64 cm) after heat treatment. Finally, the substratees with small sizes were milled with #100 abrasive paper and cleaned with non-water ethanol to eliminate the dirty spots and the oxidation layer. The cladding layer was prepared with a disk laser (TRUMPF laser HDL 4002, Ditzingen, Germany). laser experiments were performed in CW (continuous-wave) mode. The details of laser processing parameters are shown in Table 3, and the scanning orientation was along the long dimension of substrate. The experiment was repeated for three times under each group of fabrication parameters.

### 2.2. Optical System

**Figure 2:**
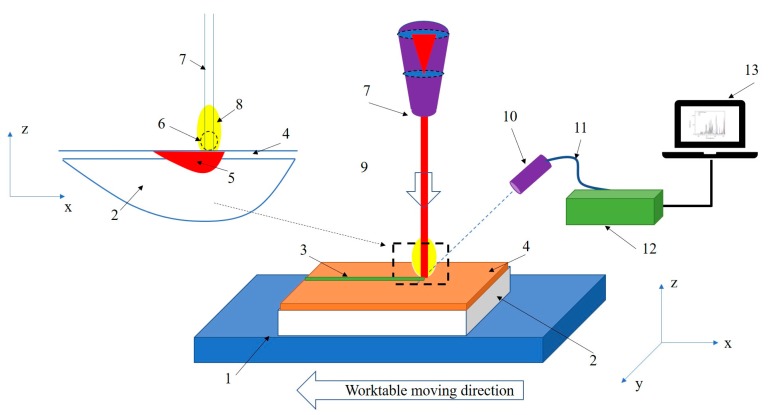
Schematic diagram of the experimental system. 1: Worktable; 2: Substrate; 3: Cladding zone; 4: Pre-placed layer; 5: Molten pool; 6: Detection area; 7: Laser beam; 8: Plasma; 9: Shield gas orientation; 10: Detection sensor; 11: Optical fiber; 12: Smart manufacture optical monitor system (SMOMS); 13: Computer with analysis software.

The experimental schematic diagram is shown in Figure 2. The SMOMS (smart manufacture optical monitor system) was designed and produced by Sensigma LLC. (Ann Arbor, MI, USA). The detected orientation was parallel to the surface of samples. The detected point for the plasma signal was 1.5 mm above the substrate and 70 mm away from the laser beam. This viewing method avoided interference from the blackbody radiation from the molten pool [15]. The spectrometer had an entrance aperture of a 10 µm width, a holographic UV grating with a groove density of 4800 per millimeter, and a 2048-element CCD (charge-coupled device)-array detector. The resolution of the spectrometer was 0.05 nm. The range of wavelength was from 340–430 nm, and the integral time for the spectra was 20 ms. Hence, the number of emission spectra can be calculated by

(1) $$N=\frac{50d}{v}$$

where *d* is the length of substrate and *v* is the scanning speed

For each sample, the number of emission spectra increased with the decline of laser scanning speed. Because the peaks intensity had large fluctuations when the laser passed the edge of substrate, considering the stability of spectra and amount of data group, only 50 spectra in the middle part of data group were selected for analyzing in next step. This meant that 150 spectra were picked up for the samples prepared with same laser parameter in total.

### 2.3. Microstructure and Composition Measurement

**Figure 3:**
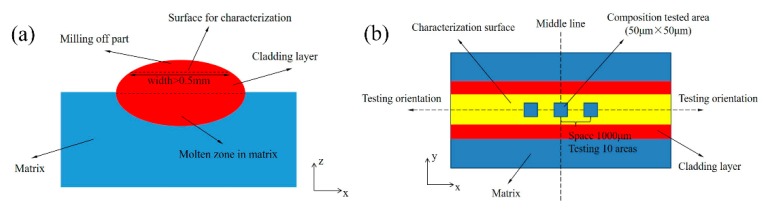
Schematic diagram of measurement of microstructure and composition of cladding layer. (**a**) sample preparation; (**b**) composition testing by area model.

The sample for the characterizations of microstructure and composition was cut from the middle of the original cladding layer, and the size was 2.54 cm × 2 cm × real height. The head of the cladding layer were grinded off where a new surface appeared on the head of cladding layer, and the schematic diagram is shown in Figure 3a. The new surface was grilled with the abrasive papers from #120 to #2000, and then it was polished with 3 μm polish paste, cleaned with anhydrous ethanol, and dried with hot air. Finally, it was etched in electrolyte solution of HCl 30 mL, H_2_O 70 mL and FeCl_3_ 50 g for 10 s. The microstructure of cladding layer was observed by SEM, and the composition was test by EDS (Tescan MIRA3 FEG SEM, USA). For the composition measurement of the second phase, the point model was used; for the average composition in cladding layer, the area model was selected. Each area size was 50 μm × 50 μm, and the tested locations were chosen from the middle to the edge of the sample. The detail of area measurement is shown in Figure 3b, and the final testing results was the average of all data.

## 3. Results and Discussion

### 3.1. Microstructure in Laser Cladding Layers

**Figure 4:**
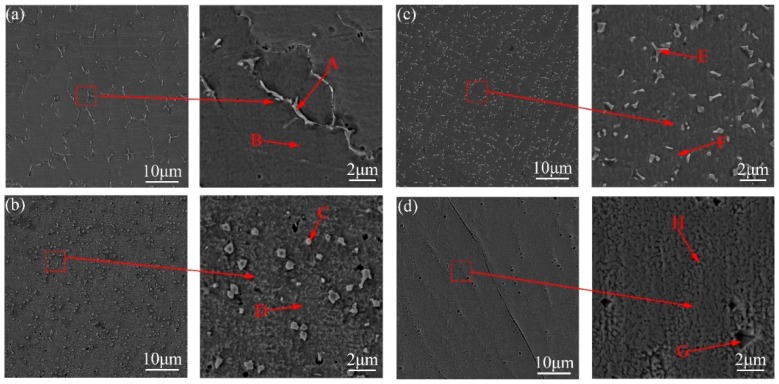
Microstructure of four kinds of laser cladding layers (1600 W, 15 mm/s and 0.8 mm of thickness). (**a**) Ni 107; (**b**) Ni 183; (**c**) Ni 202; (**d**) Ni 307.

The microstructure of different laser cladding layers is shown in Figure 4. For the number of precipitated phases, it was nearly same between Ni 183 and Ni 202. Moreover, the number in Ni 183 and Ni 202 was far bigger than that in Ni 107 and Ni 357. The shape of the precipitated phase was different among four kinds of cladding layers for Ni 107, it showed a long-bar shape, while the shape of the precipitated phase was small particle in the Ni 183 cladding layer. For Ni 202, it contained two shape types of the precipitated phase—the short-bar and the particle-like. For Ni 357, the shape of the precipitated phase was a triangle. By a comparison of precipitation location, only the phases were separated out on the inter crystal in Ni 107 cladding layer. For the other three, the phases were precipitated in the crystal.

In each kind of cladding layer, two typical locations were selected to be measured composition: One was the precipitated phase (points A, C, E, G); the other was the substrate of cladding layer (points B, D, F, H). The point testing results of composition of cladding layers (1600 W, 15 mm/s and 0.8 mm of thickness) are shown in Table 4.

### 3.2. Composition in Laser Cladding Layers

**Figure 5:**
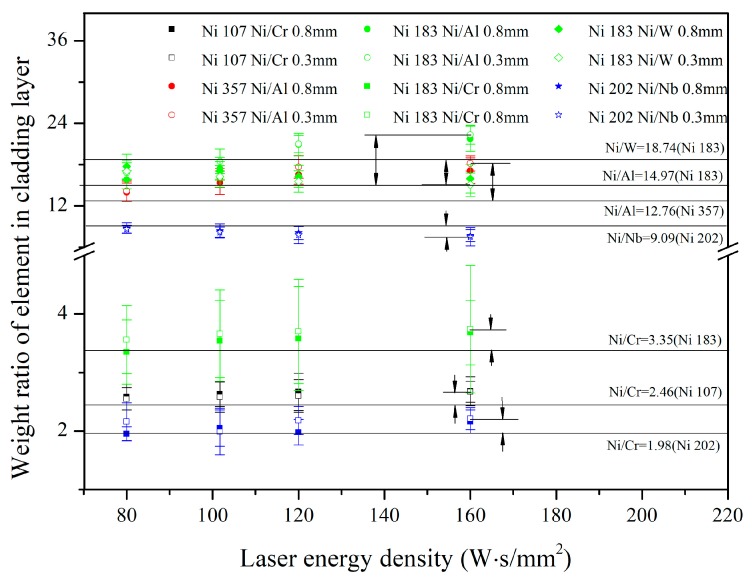
Weight ratio among different element in laser cladding layers. In this picture, the color represents the number of Ni-based alloy powders: Black-Ni 107, green-Ni 183, blue-Ni 202 and red-Ni 357. The shape of the point means weight ratio between different element: Square-Ni/Cr, circle-Ni/Al, rhombus-Ni/W and star-Ni/Nb. The empty point represents the thickness of the pre-placed powder layer (0.3) mm, and the solid point represents the thickness (0.8 mm); the parallel lines mean the weight ratios of elements in the original powders.

**Figure 6:**
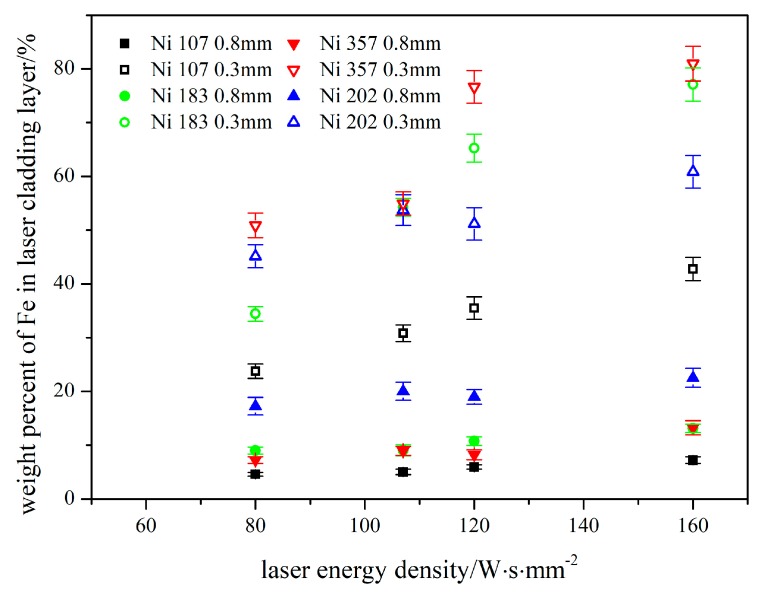
Weight percent of Fe in laser cladding layer. In this picture, the color represents the number of Ni-based alloy powders: Black-Ni 107, green-Ni 183, blue-Ni 202 and red-Ni 357. The empty point represent the thickness of pre-placed powder layer (0.3 mm), and the solid point represent the thickness (0.8 mm).

According to the measurement results shown in Table 4, it can be pointed out the precipitation of the second phase led to heterogeneous micro-composition in the cladding layers. Hence, for composition characterization of the integral cladding layer, the area model of EDS was used to test the mass percent of elements, and the variation of weight ratios among different elements versus the laser energy density is shown in Figure 5. The weight percent of the substrate (AISI 4140) elements diffused into cladding layer is shown in Figure 6.

Five elements were selected in the laser cladding layers. Besides the main substrate element (Ni), the other alloy elements could be classified into three categories: The refractory element (W and Nb), the fusible element (Al), and the element with a similar melting point to Ni (Cr). For the weight ratio between the substrate element and refractory elements (Ni/W and Ni/Nb), it showed a decreasing trend with the growth of laser energy density; for the weight ratio between two elements with similar melting points (Ni/Cr), it displayed an weakly increasing trend with the enhance of laser energy density. Correspondingly, the weight ratio between the substrate element and the fusible element (Ni/Al) also demonstrated an increasing trend, but the variation range was larger than that of Ni/Cr.

When the thickness of the pre-placed powder layer was 0.8 mm, the weight percent of Fe just showed a slight increasing trend versus the laser energy density. Moreover, the absolute increment of Fe was similar among four kinds of Ni-based cladding layers; when the thickness was 0.3 mm,for Ni 107 and Ni 202, the weight percent of Fe rose stably following the enhance of laser energy density. For Ni 183, the growth of weight percent of Fe became sharp. For Ni 357, the weight percent of Fe indicated a rapid increaset only when the laser energy density increased from 107 to 120 W·s·mm^−2^. The direct cause of the complex variation on the concentration of Fe could have been the different heating processes among four kinds of Ni-based powders. For the alloy powder used in the experiment, the thermal conductivity, refractive index, and absorptive could have varied with the concentration changes of compositions [18,19,20,21].

The direct reason for the variation of weight ratios among elements was the different ablation of elements. Depending on the Raoult’s law, the partial pressure of metal vapor can be expressed as [22]:

(2) $$p_{i}=p_{i}^{*}x_{i}$$

where $p_{i}^{*}$ is the saturated vapor pressure(atm) of element i and $x_{i}$ is the mole fraction of element i.

**Figure 7:**
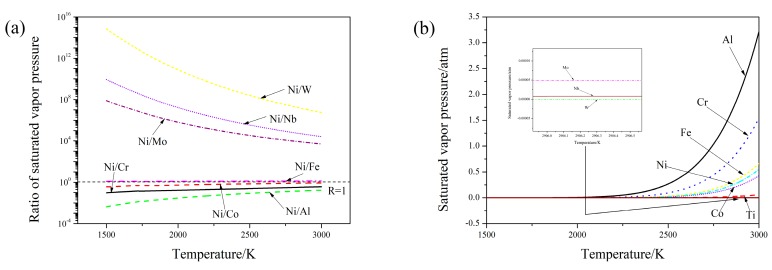
Calculation of saturated vapor pressure among different elements. (**a**) Ratio of saturated vapor pressure. (**b**) Saturated vapor pressure.

According to the data shown in References [23,24], the calculation results about saturated vapor pressure are shown in Figure 7. When the molten pool temperature was higher than 2500 K, the pressure ratios approach constants. In addition, according to the data shown in Figure 7a, the order of ablation was: Refractory element (W, Nb and Mo) < element with similar melting point of Ni (Ni, Fe, Co and Cr) < fusible element. Hence, when the laser energy density was high, the weight ratios of the elements in the cladding layer had some deviation compared by those in the original Ni-based alloy powders. Even the relative difference of pressure ratio between two elements was bigger under a lower temperaturel however, in combination with the data given by Figure 7b, when the temperature was lower than 2250 K, the absolute ablation of each element was too little, and the influence of ablation could be ignored during laser cladding process. Therefore, the weight ratios among elements was nearly same as those in the original powders when the laser energy density was low (Figure 5).

By the comprehensive analysis of Figure 5 and Figure 6, it can be pointed out that, firstly, the weight ratios of elements in Ni-based alloy powders can be influenced by the laser energy density. However, they are not affected by the quantity of elements diffused from the substrate. Secondly, the element diffused from the substrate to the cladding layer just play a dilution effect in the alloy system of the laser cladding layer. Thirdly, the correlation between the laser preparing parameters and the molten quantity of substrate was weak, which means the composition monitoring was necessary to laser cladding.

### 3.3. Spectral Analysis 

**Figure 8:**
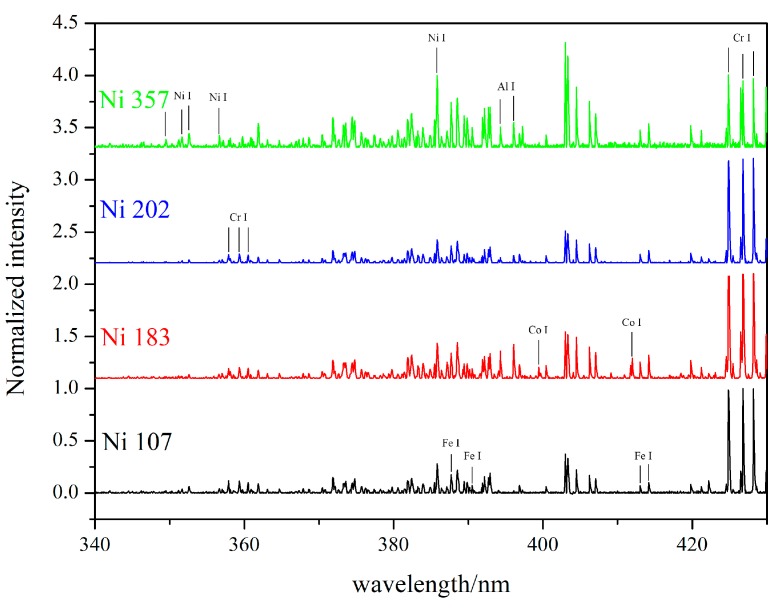
Spectra for four kinds of Ni-based laser cladding layers. (1600 W, 10 mm; the thickness is 0.8 mm).

The vaporized material diffused from the surface of molten pool and was induced by the laser beam. During the interaction, the transition of extranuclear electron generated the energy emission and formed the spectral lines with special wavelength—this is known as laser induced plasma plume [25]. Figure 8 shows the laser induced spectra for four kinds of Ni-based alloy powders during the laser cladding process. By matching with the standard wavelength of spectra lines offered in the NIST (National Institute of Standards and Technology) database [26], some of atomic spectral lines were found and are marked in Figure 8, including Fe I, Cr I, Ni I and Al I. The atomic spectral lines emitted by the refractory element, such as W, Nb and Mo, could not be observed in the spectra.

The intensity of the spectral lines can be expressed as:

(3) $$I=\frac{hcN_{0}gA}{4\pi \lambda Z}\exp\left[-\frac{E}{kT}\right]\;$$

where *h* is Planck’s constant (eV·s), *c* is the speed of light (m/s), *N_0_* is the total species population (m^−3^), *g* is the degeneracy, *A* is the transition probability or the Einstein coefficient (s^−1^), *λ* is the wavelength (m), *Z* is the partition function usually taken as the statistical weight of the ground state, *E* is the upper energy level (eV), *k* the Boltzmann constant (eV/K), and *T* the plasma temperature (K). Hence, the number of *N*_0_ can make the spectral lines emitted by refractory elements very weak due to the low number of these atoms. In addition, in the laser induced spectra, the spectral lines emitted by the ionic were not found for the low laser energy density used in the laser cladding process.

**Figure 9:**
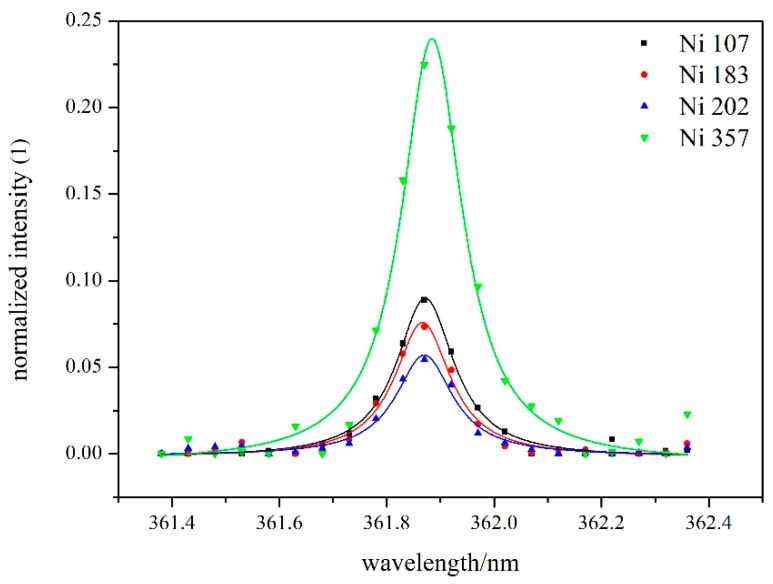
Results of Lorentz fitting of Fe I 361.877 nm.

The following spectroscopic analysis was based on the assumption that the plasma was optically thin and in a local thermodynamic equilibrium state [13,27,28,29]. Because of the Stark effect, the spectral lines were typically broadened to the Lorentz profile; hence, the original data were firstly removed the baseline, and then they were processed by the Lorentz fitting [30,31,32,33]. Finally the integral area of peak was set as the intensity of spectral lines. The typical processing results of the spectral lines are shown in Figure 9.

In this research, the calibration curves were established by the intensity ratios among different spectral lines. According to the selection standard of spectral lines reported in Reference [15], the strong and the weak lines were eliminated, and the spectral lines with small difference between upper level energy, perfect of peak profile, and well resolution were selected—these are shown in Table 5.

In consideration of the influence of the saturated vapor pressure of different elements, the component percent of elements in the plasma plume was not equal to that in the cladding layer. Hence, the intensity ratios among different elements are calibrated by:

(4) $$\varphi^{*}=\gamma \cdot \frac{I_{i}}{I_{j}}\approx \frac{N_{0}^{i}}{N_{0}^{j}-N^{\prime }}$$

(5) $$\gamma =\frac{f\left(R_{powder}\right)+be^{c\sigma }}{R_{powder}}$$

where γ is the corrected parameter, σ is the laser energy density (W·s/mm^2^), $R_{powder}$ is the weight ratio of elements in Ni-based alloy powder (1), $N_{0}^{i}$ and $N_{0}^{j}$ are the number of emitted atoms of element i and element j (m^−3^), $N^{\prime }\;$is the extra atoms caused by different saturated vapor pressure (m^−3^), and *b* and *c* are the constants relying on the elements. When the saturated vapor pressure of element j is bigger than that of elements i, γ. should be greater than 1; moreover, for two elements with similar saturated vapor pressure, γ can be regarded as 1. In this experiment, it was assumed *f*(*R_powder_*) = *dR_powder_* + *e*, γ could be deduced by the data shown in Figure 5

**Figure 10:**
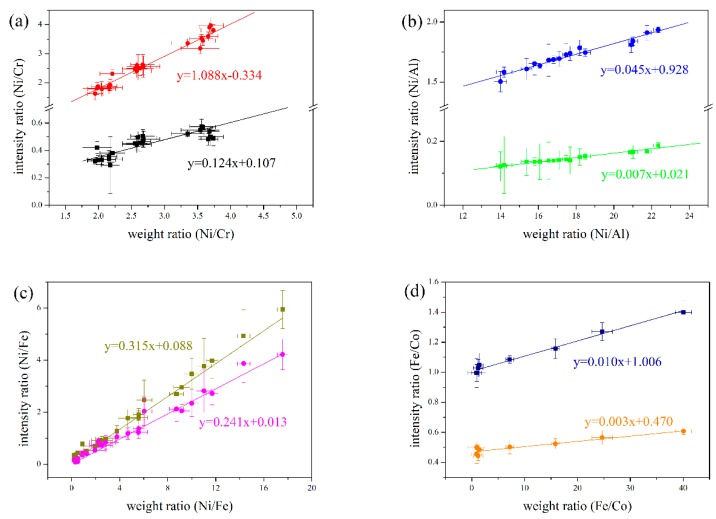
Calibration curves for different elements. (**a**) Ni I 352.454/Cr I 357.869-red, Ni I 385.830/Cr I 360.533-black; (**b**) Ni I 352.454/Al I 394.401-blue, Ni 357.186/Al I 396.152-green; (**c**) Ni I361.274/Fe I 387.250-olive, Ni I 356.637/Fe I 387.250-purple; and (**d**) Fe I 387.25/Co I 411.877-navy, Fe I 361.877/Co I 411.877-orange.

Figure 10 displays the calibration curves for different elements, including Ni/Cr, Ni/Al, Ni/Fe and Ni/Co. The weight ratio between two elements was calculated by the real component percent in four kinds of laser cladding layers, and the intensity ration between spectral lines was calculated by the data collected during the real laser cladding process.

For each group of calibration curves, the intensity ratios of spectral lines illustrated good linear correlations versus the weight ratios of components in cladding layers. However, some of calibration curves like the intensity ratios of 357.186/Al I 396.152 and Fe I 361.877/Co I 411.877 varied by the increase of element insensitively. Thus, four calibration curves (Ni I 352.454/Cr I 357.869, Ni I 352.454/Al I 394.401, Ni I361.274/Fe I 387.250 and Fe I 387.25/Co I 411.877) show good linear correlation with the weight ratios. Moreover, those showing sensitive variations with the change of composition were selected in this experiment to monitor the component percent in the laser cladding layer.

### 3.4. Component Monitoring during Laser Cladding Process

For the refractory elements in the alloy powders, e.g., W and Nb, it was hard to find the signal emitted by the atoms of these elements due to the low laser energy density used in the experiment. Thus, the weight percent of refractory elements could not be directly monitored by the emission spectroscopy. To solve this problem, the data shown in Figure 5 were used to deduce the weight percent of refractory elements. When temperature of molten pool was lower than the melting point of refractory element, the weight ratios between substrate element (Ni) and refractory element could be expressed approximatively as:

(6) $$R^{*}=\left(0.727R_{powder}+0.665\right)+\left(0.121R_{powder}+6.165\right)e^{-0.016\sigma }$$

**Figure 11:**
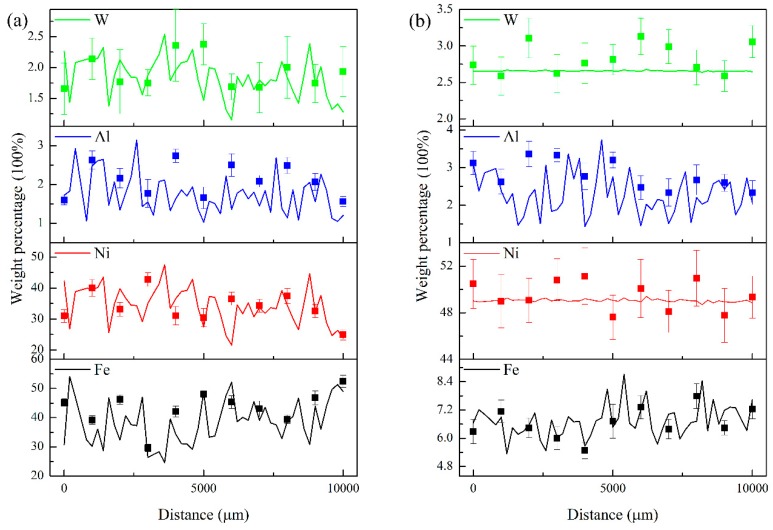
Monitoring results of weight percentage of Fe, Ni, Al and W, the points show the composition tested by EDS, and the lines show the composition predicted by the emission spectroscopy. (**a**) 800 W, 5 mm/s and 0.3 mm of thickness; (**b**) 2000 W, 20 mm/s and 0.8 mm of thickness.

To verify the accuracy of this monitoring system of component percent, two groups of processing parameters were used to fabricate the Ni 183 laser cladding layer. For the first group, the parameters were 800 W of laser power, 5 mm/s of laser scanning speed, 0.3 mm of powder thickness and 40 ms of integral time for spectra. For the second group, the parameters were 2000 W of laser power, 20 mm/s of laser scanning speed, 0.8 mm of powder thickness and 10 ms of integral time for spectra. The content monitoring results of Ni, Fe, Al and W are shown in Figure 11.

The monitoring signal of the weight percentage was smoother and more stable of Ni under the processing parameters of the second group. For Ni Fe, Al and W, the average relative deviations between the predicted concentration and the measured results by EDS were 2.26%, 7.14%, 26.25% and 6.97%, respectively. For the Fe, Ni, Al and W in the first group, the average relative deviations were 11.24%, 11.64%, 35.57% and 18.32%, respectively. By comparing the two groups of analysis results, it can be proven that the processing parameters can affect the predict results and the real elements ditribution in the laser cladding layer. The concentration variation of Fe revealed that lower scanning speed can make the depth of molten pool unstable. In the first group, the relative standard deviation (RSD) of Fe concentration was 15.81%, and the RSD in the second group was 9.05%. In addition, the signal intensity of the spectra was influenced by the concentration of the alloy vapor above the molten pool. Thus, the low laser power can decrease the heating rate of the molten pool and then increase the error rate in spectra. Furthermore, when the concentration of elements was lower than 3%, the measuring error of EDS itself could be around 10%~20%; hence, there were big differences between the predicted results by spectra analysis and the testing results by EDS. 

## 4. Conclusions

In this experiment, four kinds of Nickel-based laser cladding layers were prepared on AISI 4140 steel with different manufacturing parameters. At the same time, the emission spectra were collected and analyzed during the real-time laser cladding process. The main conclusions found in this research can be summarized as following:

1) The saturated vapor pressures were different among the elements in the cladding layers. As such, the ablation of the fusible elements was always lower than that of the refractory elements, and the real weight ratios among elements could be changed with the variation of input energy density during the laser cladding process.
2) Among the four kinds of Ni-based laser cladding layers, the dilution rate of the substrate showed complex trends following the increase of laser energy density, and the main reason causing this phenomenon was the variation of the thermophysical parameters of the alloy powders, which could affect the heating process during laser cladding. Hence, the technology of real-time monitoring of element concentration is necessary for a laser cladding process.
3) The intensity ratios of spectral lines were calibrated by a corrected parameter. The calibration curves were established by correlating the revised intensity ratios with composition concentration, and there were linear correlations between the revised intensity ratios and composition concentration in cladding layers. Finally, four calibration curves including Ni I361.274/Fe I 387.250, Ni/Al, Ni I 352.454/Cr I 357.869 and Fe I 387.25/Co I 411.877 were selected to build a monitoring system applied to laser cladding process.
4) The real-time monitoring system was used in a laser cladding process. Correspondingly, the Ni 183 laser cladding layers were prepared with different groups of processing parameters, and the composition distribution was predicted by the new monitoring system. The predicted results of composition concentration showed the low laser power or the slow scanning speed could bring a negative influence on accuracy of the monitoring system. When the depth of molten pool was stable for a component with the concertation bigger than 3 wt.%, the relative deviation of the component was lower than 8%. However, for a component with a concentration lower than 3 wt.%, the maximum relative deviation could be 26.25%. This means that the real-time monitoring system needs to be improved for the predicted accuracy on the composition with a concentration lower than 3 wt.%.

It can be found that the composition monitoring system established by emission spectroscopy is a simple and effective way to measure the composition in the laser cladding layer during real time laser manufacturing.

## Figures and Tables

**Table 1:** Composition of elements in four kinds of Ni-based alloy powders (wt.%).

Powder Number	Powder Trademark	Ni	Cr	Fe	Co	W	Mo	Nb	Ti	Al
1#	Ni 107	71.12	28.88	-	-	-	-	-	-	-
2#	Ni 183	55.84	16.66	-	8.72	2.98	4.25	-	7.8	3.73
3#	Ni 202	51.58	26.02	11.53	-	-	3.04	5.67	-	-
4#	Ni 357	92.73	-	-	-	-	-	-	-	7.27

**Table 2:** Chemical component of AISI 4140 steel (wt.%).

Element	C	Mn	Si	Mo	Cr	P	S	Fe
content	0.38–0.43	0.75–1.00	0.15–0.30	0.15–0.25	0.80–1.10	0.035 max	0.040	Balance

**Table 3:** Fabrication parameters of laser cladding layers.

Parameter Name	Experimental Value
Laser model	Continuous wavelength (1030 nm)
Focusing lens 1	Flat convex lens (CaF_2_ f = 50 mm)
Focusing lens 2	Flat convex lens (CaF_2_ f = 200 mm)
Focal spot diameter	0.6 mm
Focal spot position	4 mm above manufacturing plane
Shield gas	Ar (7.06 m^3^/min)
Laser power	1200/1600 W
Laser scanning speed	10/15 mm/s
Laser beam size	1 mm
Pre-placed powder thickness	0.3/0.8 mm
Repetition times	3

**Table 4:** Results of point measurement of EDS in four kinds of Ni-based cladding layers (wt.%).

Point Name	Point Location	Ni	Cr	Fe	Co	W	Mo	Nb	Ti	Al
A	precipitated phase	32.24	38.67	29.09	-	-	-	-	-	-
B	Substrate	73.04	23.66	2.37	-	-	-	-	-	-
C	precipitated phase	2.18	4.98	0.59	0.04	22.29	16.72	-	47.81	0.1
D	Substrate	48.06	18.62	12.70	7.71	3.12	3.25	-	3.89	2.66
E	precipitated phase	12.06	9.32	3.67	-	-	1.74	64.42	8.38	-
F	Substrate	53.87	25.07	13.55	-	-	3.09	3.80	0.25	-
G	precipitated phase	83.17	-	11.54						5.29
H	Substrate	81.94		12.57						5.49

**Table 5:** Parameters of spectral lines for the lines of component calibration.

Element	Wavelength (nm)	g_ik_A (s^−1^)	E_i_ (eV)	E_k_ (eV)
Fe I	361.877	505,000,000	0.990	4.416
Fe I	387.250	52,500,000	0.990	4.191
Cr I	357.869	1,330,000,000	0.000	3.464
Cr I	360.533	810,000,000	0.000	3.438
Ni I	352.454	500,000,000	0.025	3.542
Ni I	356.637	280,000,000	0.423	3.899
Ni I	357.186	36,000,000	0.165	3.636
Ni I	361.274	21,000,000	0.275	3.706
Ni I	385.830	48,000,000	0.423	3.636
Co I	411.877	130,000,000	1.049	4.059
Al I	394.401	49,900,000	0.000	3.143
Al I	396.152	98,500,000	0.014	3.143

## References

[1] Kusinski J., Kac S., Kopia A., Radziszewska A., Rozmus-Górnikowska M., Major B., Major L., Marczak J., Lisiecki A. (2012). Laser modification of the materials surface layer—A review paper. Bull. Pol. Acad. Sci. Tech. Sci..

[2] Mali H.S., Unune D.R., Beddows C. (2017). Machinability of Nickel-Based Superalloys: An Overview. Reference Module in Materials Science and Materials Engineering.

[3] Thellaputta G.R., Chandra P.S., Rao C.S.P. (2017). Machinability of Nickel Based Superalloys: A Review. Mater. Today Proc..

[4] Muro M., Leunda J., Artola G., Soriano C. (2019). Microstructural Tuning of a Laser-Cladding Layer by Means of a Mix of Commercial Inconel 625 and AISI H13 Powders. Materials.

[5] Chen Y., Guo Y., Lu B., Xu M., Xu J. (2017). Microstructure and Properties of the Interface Area in the Laser Cladded Ni Based Coatings on the 1Cr10Mo1NiWVNbN Steel. Metals.

[6] Heigel J.C., Michaleris P., Palmer T.A. (2015). In situ monitoring and characterization of distortion during laser cladding of Inconel^®^ 625. J. Mater. Process. Technol..

[7] Dinda G.P., Dasgupta A.K., Mazumder J. (2012). Texture control during laser deposition of nickel-based superalloy. Scr. Mater..

[8] Verdi D., Garrido M.A., Múnez C.J., Poza P. (2014). Mechanical properties of Inconel 625 laser cladded coatings: Depth sensing indentation analysis. Mater. Sci. Eng. A.

[9] Cremers D.A., Multari R.A., Knight A.K. (2009). Laser-induced Breakdown Spectroscopy. Appl. Spectrosc. Rev..

[10] Aragon C., Aguilera J.A., Penalba F. (1999). Improvements in Quantitative Analysis of Steel Composition by Laser-Induced Breakdown Spectroscopy at Atmospheric Pressure Using an Infrared Nd: YAG Laser. Appl. Spectrosc..

[11] Ciucci A., Corsi M., Palleschi V., Rastelli S., Salvetti A., Tognoni E. (1999). New Procedure for Quantitative Elemental Analysis by Laser-Induced Plasma Spectroscopy. Appl. Spectrosc..

[12] Death D.L., Cunningham A.P., Pollard L.J. (2008). Multi-element analysis of iron ore pellets by Laser-induced Breakdown Spectroscopy and Principal Components Regression. Spectrochim. Acta Part B At. Spectrosc..

[13] Yaroshchyk P., Body D., Morrison R.J.S., Chadwick B.L. (2006). A semi-quantitative standard-less analysis method for laser-induced breakdown spectroscopy. Spectrochim. Acta Part B At. Spectrosc..

[14] Liu W., Liu S., Ma J., Kovacevic R. (2014). Real-time monitoring of the laser hot-wire welding process. Opt. Laser Technol..

[15] Song L., Mazumder J. (2012). Real Time Cr Measurement Using Optical Emission Spectroscopy during Direct Metal Deposition Process. IEEE Sens. J..

[16] Song L., Wang C., Mazumder J. Identification of phase transformation using optical emission spectroscopy for direct metal deposition process. Proceedings of the High Power Laser Materials Processing: Lasers, Beam Delivery, Diagnostics, and Applications.

[17] Song L., Huang W., Han X., Mazumder J. (2017). Real-Time Composition Monitoring Using Support Vector Regression of Laser-Induced Plasma for Laser Additive Manufacturing. IEEE Trans. Ind. Electron..

[18] Saunders N., Miodownik A.P., Schillé J.P. (2004). Modelling of the thermo-physical and physical properties for solidification of Ni-based superalloys. J. Mater. Sci..

[19] Seifter A., Pottlacher G., Jäger H., Groboth G., Kaschnitz E. (1998). Measurements of thermophysical properties of solid and liquid Fe-Ni alloys. Ber. Bunsenges. Phys. Chem..

[20] International A.S.M., Rights A. (2013). ASM Specialty Handbook: Nickel, Cobalt, and Their Alloys.

[21] Dobrovska J., Zla S., Kavicka F., Smetana B., Vodarek V. (2012). Study of Thermo-Physical Properties of Selected Nickel-Based Superalloys with Use of DTA Method. Proceedings of the ASME 2012 11th Biennial Conference on Engineering Systems Design and Analysis (ESDA 2012).

[22] Atkins P., de Paula J. (2006). Physical Chemistry.

[23] Lide D.R. (2004). CRC Handbook of Chemistry and Physics.

[24] Alcock C.B., Itkin V.P., Horrigan M.K. (1984). Vapour Pressure Equations for the Metallic Elements: 298–2500 K. Can. Metall. Q..

[25] Noll R. (2012). Laser-Induced Breakdown Spectroscopy.

[26] NIST Atomic Spectra Database Online (U.S Department of Commerce 2016). https://physics.nist.gov/PhysRefData/ASD/lines_form.html.

[27] Shin J., Mazumder J. (2016). Plasma diagnostics using optical emission spectroscopy in laser drilling process. J. Laser Appl..

[28] Capitelli M., Colonna G., Gorse C., D’angola A. (2000). Transport properties of high temperature air in local thermodynamic equilibrium. Eur. Phys. J. D.

[29] Hahn D.W., Omenetto N. (2010). Laser-Induced Breakdown Spectroscopy (LIBS), Part I: Review of Basic Diagnostics and Plasma–Particle Interactions: Still-Challenging Issues Within the Analytical Plasma Community. Appl. Spectrosc..

[30] Lesage A. (2009). Experimental Stark widths and shifts for spectral lines of neutral and ionized atoms A critical review of selected data for the period 2001–2007. New Astron. Rev..

[31] Cremers D.A., Radziemski L.J., Cremers D.A., Radziemski L.J. (2006). Basics of the LIBS Plasma. Handbook of Laser-Induced Breakdown Spectroscopy.

[32] Singh J.P., Thakur S.N., Elsevier Science (2007). Thakur Atomic Emission Spectroscopy. Laser-Induced Breakdown Spectroscopy.

[33] Cressault Y., Hannachi R., Teulet P., Gleizes A., Gonnet J.-P., Battandier J.-Y. (2008). Influence of metallic vapours on the properties of air thermal plasmas. Plasma Sources Sci. Technol..

